# Unraveling an Enigmatic Triad: A Case Report of Concurrent Neurosyphilis, Ocular Syphilis, and Otosyphilis in a Patient with HIV

**DOI:** 10.5811/cpcem.21309

**Published:** 2025-02-26

**Authors:** Peter Njouda Shitebongnju, Alexander A. Bobrov

**Affiliations:** St. Rita’s Medical Center, Division of Emergency Medicine, Lima, Ohio

**Keywords:** case report, HIV, neurosyphilis, ocular syphilis, otosyphilis

## Abstract

**Introduction:**

Patients with HIV disease, regardless of the phase of infection, can present with overlapping stages and less distinct signs of syphilis, complicating diagnosis and treatment. *Treponema pallidum*, the bacterium responsible for syphilis, can lead to neurosyphilis, ocular syphilis, and otosyphilis when left untreated. Therefore, early detection of syphilis coinfection in HIV patients and timely treatment have demonstrated prompt improvement of symptoms, mitigating the risk of serious complications.

**Case Report:**

We report the case of a 39-year-old previously incarcerated male with a significant history of HIV on antiretroviral therapy and previous methamphetamine abuse referred to the emergency department from an ophthalmologist with a diagnosis of anterior uveitis and papilledema. The patient reported experiencing blurry vision, tinnitus, and memory difficulties. A thorough history and physical examination, along with diagnostic procedures, including lumbar puncture and cerebrospinal fluid analysis, corroborated the diagnosis of neurosyphilis with otic and ocular involvement. The patient underwent a 14-day course of intravenous aqueous crystalline penicillin G, resulting in symptom improvement.

**Conclusion:**

Given the prevalence of syphilis and its diverse manifestations, clinicians must maintain a high index of suspicion in patients who engage in high-risk behaviors to facilitate early diagnosis and treatment, which are crucial for optimal outcomes and enhanced prognosis.

## INTRODUCTION

Patients with HIV, irrespective of the stage of infection, can present with atypical features and potentially overlapping stages of syphilis co-infection. Once exposed, the primary stage of syphilis emerges typically between 9–90 days with the hallmark syphilitic chancre and other primary lesions in the genitalia.^1^ Secondary syphilis develops subsequently over the next months, often marked by a multitude of symptoms including mucocutaneous eruptions, generalized lymphadenopathy, condyloma lata, and nephrotic syndrome.^2^ Latent syphilis is a phase of the disease characterized by a lack of overt symptoms, which presents a diagnostic challenge due to the absence of typical clinical manifestations associated with secondary syphilis. The latent stage can be further subdivided into early and late latency, with early latency marked by subtle signs and symptoms that may easily be overlooked, while late latency is defined by the complete absence of primary and secondary symptoms. Although rare, tertiary syphilis may occur years to decades after infection and is associated with potentially devastating complications, such as aortitis, coronary artery disease, and late neurological issues.^3^ Given the spirochete’s ability to rapidly invade multiple tissues in the early stages of syphilis, it is crucial to maintain a high level of suspicion for neurosyphilis in immunocompromised patients.

## CASE REPORT

A 39-year-old male with a history of HIV, prediabetes, and umbilical hernia presented to the emergency department (ED) complaining of bilateral, non-painful, non-pruritic eye redness with mild tearing, headache, and tinnitus with decreased hearing for three weeks. The patient reported persistent visual disturbances, characterized by haziness in the right visual field and black floaters with a diagonal line in the left visual field. Additionally, the patient’s partner reported that the patient began to exhibit signs of confusion and difficulty hearing around the same time. Approximately a week before the ED visit, the patient had presented to an urgent care and was diagnosed with bacterial conjunctivitis, but the prescribed ophthalmic drops failed to alleviate his symptoms. He subsequently visited an ophthalmologist, who reported evidence of papilledema as well as anterior uveitis, and referred the patient to the ED for further evaluation.

The patient disclosed he had recently experienced joint and muscle pain that he attributed to his antiretroviral therapy (ART) regimen, which had prompted him to discontinue his medication for approximately three weeks prior to onset of symptoms. The patient’s social history included tobacco and methamphetamine use, previous incarceration, and an HIV diagnosis made during incarceration. Importantly, the patient’s ART resulted in a significant improvement in his HIV viral load (from 268,000 per milliliter [mL] down to 30 copies/mL) (reference range: 0 copies/mL) and cluster of differentiation 4 (CD4+) cell count (from 142 per microliter [μL] up to 508 cells/μL) (430–1800 cells/μL) within six months of treatment initiation. The patient reported being in a monogamous relationship with a woman since his release and denied any history of sex with men, intravenous drug use, or recent travel. His primary care physician monitored his health and ART response. After a thorough review, the patient endorsed night sweats, subjective fever, and desquamation of the soles of his feet.

Upon arrival to the ED, his initial vital signs were blood pressure 120/82 millimeters of mercury, heart rate 95 beats per minute, respiratory rate 19 breaths per minute, temperature 97.9 °Fahrenheit, saturation of peripheral oxygen 100% room air, and body mass index 26.39 kilograms per cubic meter (kg/m^3^) (18.5–24.9 kg/m^3^). Physical examination was remarkable for a male who appeared generally healthy but was experiencing mild visual distress due to recent use of ocular dilating medications at ophthalmologic appointment but no photophobia. There was moderate bilateral conjunctival injection, pale sclerae, and no discharge. Pupils were dilated, equal, round, and reactive with direct and consensual responses. Extraocular movements were intact. Visual acuity testing was 20/50 in the right eye, but he was only able to count fingers with the left eye, reporting severe blurriness. Peripheral visual field testing was intact bilaterally. Cardiac exam was unremarkable with normal rate and rhythm without murmurs. Equal and bilateral lung sounds were appreciated without wheezing, crackles, or rhonchi. There was no nuchal rigidity present. There was mild non-erythematous, non-pruritic, whitish desquamation on the soles of his feet, more on the right. There was no focal weakness in any extremity, and he had a normal gait. Cranial nerves II-XII were grossly intact.

**CPC-EM Capsule**

What do we already know about this clinical entity?

*Prompt recognition and treatment of neurosyphilis, ocular syphilis, and otosyphilis are crucial to prevent complications*.

What makes this presentation of disease reportable?

*This case of an uncommon triad of syphilis in an HIV patient highlights the need to consider syphilitic infections in complex cases with seemingly unrelated symptoms*.

What is the major learning point?

*Clinicians should maintain a broad differential diagnosis and multidisciplinary collaboration when assessing HIV patients with complex neurological symptoms*.

How might this improve emergency medicine practice?

*Recognizing this rare triad of syphilitic infections in the era of a surge in syphilitic infections globally can improve clinical vigilance*.

Comprehensive metabolic panel showed normal electrolytes, kidney function, and transaminases with mildly elevated glucose. Serum non-treponemal and fluorescent treponemal antibody testing were reactive, with a subsequent serum reactive quantitative and confirmatory Venereal Disease Research Laboratory (VDRL) with a titer of 1:1024, which resulted during patient’s admission. Lumbar puncture was technically challenging but successfully performed in the ED without a measured opening pressure. Cerebrospinal fluid (CSF) was clear in appearance with mild pleocytosis. Cerebrospinal fluid VDRL quantitative and confirmatory tests were reactive, confirming diagnosis. FilmArrayME meningoencephalitis CSF panel was negative. The CSF culture ultimately did not grow any organisms and did not demonstrate presence of malignant cells. Computed tomography of the head was unremarkable for acute findings. Magnetic resonance imaging of the brain, requested by the referring consultant, demonstrated small filling defects within the left transverse sinus.[Table t1-cpcem-9-173]

The patient was admitted for treatment of neurosyphilis with ocular and otic involvements and started on penicillin G and antiretrovirals. The infectious disease specialist recommended penicillin G intravenous (IV) treatment at a dosage of 24 million units per day for 14 days, administered every four hours during hospitalization and a continuous infusion once the patient was discharged. The patient’s home dose of bictegravir-emtricitabine-tenofovir (50-200-25 mg per tablet once daily) was continued, and erythromycin ophthalmic ointment was initiated. Magnetic resonance imaging findings were interpreted by the interventional neurologist as either arachnoid granulation or non-obstructive thrombus, neither of which would explain the papilledema. Consequently, a daily, low-dose aspirin with subsequent outpatient follow-up in six months was recommended.

After three days of treatment, the patient reported improvement in his visual symptoms, indicating that the black line and floater were no longer affecting his vision. Additionally, the patient noted a reduction in his tinnitus and reported overall improvement. A follow-up appointment four days after hospitalization revealed that the patient had begun to experience hair loss, but he reported continued improvement in his vision, particularly in the left eye. On day 14, the patient visited his infectious disease physician after completing his IV benzylpenicillin treatment. The patient was advised to continue with antiretroviral therapy and to follow up with his primary care physician and neurologist as scheduled.

## DISCUSSION

*Treponema pallidum*, the causative agent of syphilis, is an adept infiltrator, capable of eluding the body’s immune defenses, sequestering itself in a multitude of tissues, and even breaching the blood-brain barrier prior to the onset of initial symptoms.^4^ It can be transmitted through intimate physical contact with an individual who harbors a syphilis-related skin lesion, which often arises through sexual intercourse. Although the patient suspected that he acquired the infection through an accidental exposure of fecal-ocular material during plumbing work prior to the onset of symptoms, this mode of transmission seems unlikely due to the limited survival of *T pallidum* outside the human host. Attempts to culture this organism in vitro have been mostly unsuccessful, with few documented success cultures performed within tissue culture systems.^5^

If untreated, the insidious pathogen can affect multiple systems including cardiovascular, neurological, otic, ocular, and immunological systems. Timely treatment, however, has shown the potential to reverse certain debilitating injuries inflicted by the infection, emphasizing the importance of prompt recognition and intervention.^6^ This patient’s experience underscores the significance of early detection and treatment in the battle against syphilis, which has been on the rise in the United States since 2001. According to the US Centers for Disease Control and Prevention (CDC), there has been a surge in syphilis infections among specific demographic groups between 2018–2022, with a disproportionate increase of 146.7% in incidence among men who have sex with women, compared to a comparatively lower 6.6% increase among men who have sex with men.^7^ These findings highlight the vulnerability of individuals engaging in high-risk sexual behaviors, including those with HIV, emphasizing the need for targeted interventions and tailored public health strategies to curb the spread of syphilis within the population.

To avoid premature closure, papilledema and uveitis reported by the ophthalmologist, in conjunction with the patient’s reported symptoms, required further investigation. Differential diagnoses were extensive, including HIV-associated meningitis or encephalitis with pathogens including cytomegalovirus, Epstein-Barr virus, tuberculosis, syphilis, toxoplasmosis, Cryptococcus, herpes simplex virus, aspergillus; cerebral venous sinus thrombosis; lymphoma; HIV-associated encephalopathy; HIV-associated neurocognitive disorder; idiopathic intracranial hypertension; typical and atypical causes of conjunctivitis and uveitis including allergic, bacterial, and viral.

While regular screenings are recommended for high-risk populations,^8^ this is often challenging to execute due to a combination of factors, including worsening difficulty in access to primary care with the COVID-19 outbreak, difficulty of notifying partners particularly in an era of online dating and sexual encounters,^9^ and insufficient resources to tackle the crisis. Interestingly, the patient’s last documented syphilis screening was 19 months prior to this hospital visit, supporting the challenges in screening this vulnerable population.

Laboratory diagnostics of syphilis remain challenging. Treponemal and non-treponemal testing, both implied in traditional and reverse-sequence screening algorithms, are used to aid in the diagnosis of syphilis. Serological treponeme-specific tests such as fluorescent treponemal antibody absorption (FTA-ABS) used to rule out neurosyphilis are reliably reactive in patients with neurosyphilis including late-stage neurosyphilis.^3^ Although CSF VDRL is the gold standard for the diagnosis of neurosyphilis, its low sensitivity and high false negative rate of up to 70% makes it an unreliable measure to rule out the disease. Similarly, CSF fluid FTA-ABS test has limitations and may not be a definitive diagnostic tool.

Treatment of syphilis depends on the stage of the disease and the organs involved per CDC guidelines. Early syphilis (primary, secondary, or early latent) is treated with a single dose of penicillin. Late latent or syphilis of unknown duration requires three doses of penicillin. Otosyphilis, ocular syphilis, or neurosyphilis are treated with aqueous crystalline penicillin G 18–24 million units per day, administered as 3–4 million units by IV every four hours, or by continuous infusion, for 10–14 days.^7^

## CONCLUSION

A high suspicion of neurosyphilis should be maintained in immunocompromised patients who present with neurological symptoms, irrespective of recent, non-treponemal screening findings. A full workup, using the appropriate screening algorithm, is imperative to rule out this organism in symptomatic patients. Accurate staging and adherence to the treatment recommendations provided by the CDC have demonstrated favorable treatment outcomes and improved prognosis.

## Figures and Tables

**Table t1-cpcem-9-173:** Lab findings of patient hospitalized for treatment of neurosyphilis with ocular and otic involvements.

Blood count	Basic metabolic panel	Liver function test	Microbiology (serum-PCR)	Cerebrospinal fluid
Hemoglobin 12.3 g/dL (Ref: 14–18)	Sodium 138 mEq/L (Ref: 135–145)	ALT 25 U/L (Ref: 11–66)	RPR positive	Color: clear
Hematocrit 39.3% (Ref: 42.0–52.0)	Potassium 4.2 mEq/L (Ref: 3.5–5.2)	AST 28 U/L (Ref: 5–40)	VDRL 1:1024	RBC 11 mm^3^ (Ref: 0)
WBC 6.1 thou/mm^3^ (Ref: 4.8–10.8)	Chloride 103 mEq/L (Ref: 98–111)	ALP 106 U/L (Ref: 38–126)	HIV-1 RNA 112 cpy/mL (Ref: 0)	Nucleated cells 11/ mm^3^ (Ref: 0–5)
Platelets 263 thou/mm^3^ (Ref: 130–400)	Bicarbonate 23 mEq/L (Ref: 23–33)	Bilirubin, total 0.3 mg/dL (Ref: 0.3–1.2)	EBV negative	Lymphocytes 93% (Ref: 0–90)
Lactate 1.2 mmol/L (Ref: 0.5–1.9)	BUN 12 mg/dL (Ref: 7–22)	Albumin 3.6 g/dL (Ref: 3.5–5.1)	TB Quant negative	Glucose 65 mg/dL (Ref: 40–80)
Absolute CD4+ 190/μL (Ref: 430–1800)	Creatinine 1.0 mg/dL (Ref: 0.4–1.2)	Protein, total 8.3 g/dL (Ref: 6.1–8.0)	Toxoplasma IgM less than 3.0 AU/mL (Ref: less than 8)	Protein 109 mg/dL (Ref: 12–60)
	Glucose 120 mg/dL (Ref: 70–108)		CMV negative	Lactate 2.3 mmol/L (Ref: 0.5–2.2)

*μL*, microliter; *U/L*, units per liter; *thou/mm^3^*, thousand per cubic millimeters; *mmol*, millimoles; *mg/dL*, milligrams per deciliter; *g*, grams; *mEq*, milliequivalents; *cpy/mL*, copies per milliliter; *ALP*, alkaline phosphatase; *ALT*, alanine aminotransferase; *AST*, aspartate aminotransferase; *AU*, antibody units; *BUN*, blood urea nitrogen; *CD4+*, cluster of differentiation 4; *CMV*, cytomegalovirus; *EBV*, Epstein-Barr virus; *RNA*, ribonucleotide; *IgM*, immunoglobulin M; *PCR*, polymerase chain reaction; *RBC*, red blood cells; *Ref*, reference range; *RPR*, rapid plasma reagin; *TB Quant*, tuberculosis QuantiFERON; *VDRL*, venereal disease research laboratory; *WBC*, white blood count.
